# Age and seasonal variation of serum vitamin D levels in healthy school children and adolescents

**DOI:** 10.1186/1687-9856-2013-S1-P159

**Published:** 2013-10-03

**Authors:** Min Sun Kim, You Jean Yang, Pyoung Han Hwang, Dae-Yeol Lee

**Affiliations:** 1 Department of Pediatrics, Chonbuk National University Medical School, Jeonju, Korea

## Purpose

Vitamin D is an important fat-soluble vitamin that functions as a prohornone and affects bone mineralization and calcium homeostasis. In this study, we measured serum vitamin D levels in healthy school children and adolescents, and evaluated the prevalence of vitamin D defficiency, and its correlation with age, season and other clinical parameters.

## Methods

We included 706 school children and adolescents aged 7 to 18 years (434 girls and 272 boys) from March 2011 to February 2012. We excluded subjects with any acute or chronic diseases. None of subjects were taking calcium or vitamin D supplements. They were classified according to age (elementary school, 7-12 years old, n=565; middle school, 13-15 years old, n=75; high school, 16-18 years old, n=36), sex (boys, n=272; girls, n=434) and season (spring, March to May, n=267; summer, June to August, n=106; fall, September to November, n=139; winter, December to February, n=194). We performed anthropometric measurement and laboratory tests including fasting lipid profile (cholesterol, triglyceride and LDL cholesterol), ALP, Ca, P and serum 25OHD_3_ level. Vitamin D deficiency were defined as a serum 25OHD_3_ level lesser than 20 ng/mL.

## Results

1) In total 706 subjects, mean serum vitamin D level was 16.30 ± 6.10 ng/mL, and prevalence of vitamin D defficiency was 74.5%. The serum vitamin D level was negatively correlated with age, serum estradiol and LH values.

2) Mean serum vitamin D levels were 16.65 ± 6.07 ng/mL in elementary school group, 15.23 ± 6.37 ng/mL in middle school group, and 12.65 ± 5.56 ng/mL in high school group. The prevalence of vitamin D deficiency in 3 aged groups was 72.9% in elementary school group, 78.7% in middle school group, and 91.7% in high school group. However, in elementary school group, there was no significant difference in vitamin D levels between low grade (7-9 years old) and high grade subjects (10-12 years old).

3) The level of serum vitamin D was significantly higher in summer (20.99 ± 6.40 ng/mL) and fall (19.11 ± 6.11 ng/mL) than in spring (15.46 ± 5.22 ng/mL) and winter (12.88 ± 4.37 ng/mL). The prevalence of vitamin D deficiency was 93.8% in winter and 49.1% in summer.

4) Mean serum vitamin D level was significantly lower in girls (15.73 ± 5.71 ng/mL) than in boys (17.20 ± 6.59 ng/mL). The prevalence of vitamin D deficiency was 68.8% in boys and 78.1% in girls.

## Conclusion

The prevalence of vitamin D deficiency in healthy school children and adolescent was very high, especially in high school adolescents and winter season. These findings suggest that adequate outdoor activity and vitamin D supplements should be necessary for school children and adolescents.

